# Douleurs induites par les soins: la réalité au Centre Hospitalier Universitaire de Befelatanana Antananarivo, Madagascar

**DOI:** 10.11604/pamj.2014.19.358.5333

**Published:** 2014-12-08

**Authors:** Ernestho-Ghoud Indretsy Mahavivola, Razanaparany Miarisoa Mireille Olivah, Dodo Mihary, Rakotoharivelo Hendriniaina, Randriamboavonjy Rado Lalao, Rakotonirainy Oliva Henintsoa, Rapelanoro Rabenja Fahafahantsoa

**Affiliations:** 1 Unité de Soins, de Formation et de la Recherche en Rhumato-Dermatologie, Hôpital Joseph Raseta Befelatanana, Antananarivo, Madagascar; 2 Laboratoire d'Appui aux Recherches et Technologies de l'Information et de la Communication, Faculté de Médecine d'Antananarivo, Madagascar

**Keywords:** Douleurs induites par les soins, épidémiologie, Antananarivo, Madagascar, care induced pain, epidemiology, Antananarivo, Madagascar

## Abstract

La douleur induite par les soins correspond à la douleur survenant lors des actes à visé diagnostique et/ou thérapeutique. A notre connaissance, nous n'avons pas encore des données disponibles pour les douleurs induites par les soins à l'Hôpital de Befelatanana. Nos objectifs étaient de décrire le profil épidémiologique de la douleur induite par les soins, d'identifier les principaux facteurs influençant sur l'intensité de la douleur et leurs retentissements chez les patients. Il s'agissait d'une étude rétrospective, transversale type un jour donné menée dans les douze services de Médecines au Centre Hospitalier Universitaire de Befelatanana en Novembre 2013. Cent deux patients ont été retenus dans l’étude et trois cent vingt trois actes douloureux étaient enregistrés. La fréquence de la douleur induite par les soins était de 69,86%. Le genre féminin prédominait dans 52% des cas (n = 53) avec un sex-ratio à 0,92. L’âge moyen était de 46 ans. Les ponctions vasculaires étaient l'acte prédominant dans 49,54% (n = 109) des cas. Les infirmiers réalisaient les soins dans 47,05% (n = 48) des cas. L'information verbale était la mesure préventive utilisée dans 57,84% des cas (n = 59). Le transport par marche à pied et au dos représentait 16,67% des cas (n = 17). Les patients naïfs des gestes étaient plus anxieux. Ces patients gardaient de mauvais souvenir dans 64,71% des cas (n = 66). La fréquence de douleur induite par les soins était trop élevée. Un effort important est nécessaire pour réduire la douleur induite par les soins

## Introduction

Les douleurs « induites» sont des douleurs de courte durée, déclenchées par les soins médicaux dans des circonstances de survenue prévisible et donc susceptible d’être prévenue par des mesures adaptées [1]. A notre connaissance, aucune étude sur les douleurs induites par les soins n'a jamais été évaluée au CHU de Befelatanana. La disponibilité de telles données permettrait d'améliorer la prise en charge des malades. Nos objectifs étaient de décrire le profil épidémiologique de la douleur induite par les soins et d'identifier les principaux facteurs influençant sur l'intensité de la douleur ainsi que son éventuel retentissement chez les patients hospitalisés.

## Méthodes

Il s'agissait d'une étude rétrospective, descriptive et transversale type un jour donné, réalisée dans des services de Médecine en dehors des Urgences au CHU de Befelatanana sur une durée de deux semaines en Novembre 2013. Les patients étaient sélectionnés aléatoirement pour un entretien sur les épisodes ou évènements douloureux qui s’étaient produits durant son hospitalisation. Les patients adultes hospitalisés depuis plus de 24 heures, qui avaient bénéficié d'au moins un soin médical et étaient capable de répondre aux questionnaires, étaient inclus dans ce travail. Nous avions exclus les patients dans l'impossibilité d'auto-évaluer sa douleur (barrière de la langue, troubles de la vigilance, déficit visuel). Nous avons analysé les paramètres démographiques, les caractéristiques des soins, l'intensité de la douleur induite par les soins, les facteurs influençant sur l'intensité de la douleur et les retentissements. Nous avons classé les caractéristiques des soins médicaux en sept procédures: ponctions vasculaires (prélèvement sanguin, pose d'un cathéter), ponctions non vasculaires (ponction lombaire, pleurale, ascite, articulaire, médullaire...), gestes à visées diagnostiques ou thérapeutiques (fibroscopie digestive haute, fibroscopie bronchique, biopsies...), autres gestes invasives (pose d'une sonde urinaire, sonde naso-gastrique, tubage), traitements par injection (intraveineuse directe, intramusculaire, sous-cutané, infiltration), autres soins thérapeutiques (pansements, mobilisation), transports (brancard, chaise roulante). Les soins étaient réalisés par les personnels permanents des services et les stagiaires.

Les patients étaient considérés « douloureux» s'ils avaient ressenti des épisodes douloureux au moment ou après les soins. Nous avons utilisé l'Echelle Verbale Simplifiée pour évaluer l'intensité de la douleur. Cette échelle a été testée et validée chez les patients Malgaches dans les douleurs ostéo-articulaires pour sa facilité d'utilisation. L'intensité des douleurs perçues étaient reparties comme suit: douleur faible= 1, modérée= 2, sévère= 3 et extrêmement sévère= 4. Par contre, l'intensité de l'anxiété était mesurée par l'Echelle Numérique de 0 à 4 (0= absence d'anxiété, 1= anxiété faible, 2= anxiété modérée, 3= anxiété intense, 4= anxiété extrêmement intense). Les données ont été recueillies manuellement par un seul enquêteur à travers les questionnaires préalablement testé. L'analyse statistique était réalisée à l'aide du logiciel Epi info 7. Le seuil de significativité retenu était une valeur de p ≤ 0,05. Dans un premier temps, une analyse descriptive a été réalisée pour montrer les proportions, puis pour les comparer, un test de chi2 de Pearson a été réalisé. Si les conditions d'application n’étaient pas possibles, un test exact de Fisher a été appliqué.

## Résultats

Nous avions interrogé 146 patients hospitalisés en un jour donné dans notre centre sur 608 patients hospitalisés. Cent deux patients respectaient nos critères. La prévalence de la douleur induite par les soins était de 69,86%. Soixante dix sept pourcent signalaient encore l'existence d'une douleur au cours des 24 heures après l'acte. Le genre féminin prédominait dans 51,96% (n = 53) (p = 0,58), avec un sex-ratio à 0,92. La moyenne d’âge était de 46 ans avec un extrême allant de 16 ans à 82 ans. Le nombre des douleurs induites par les soins augmentaient progressivement entre l’âge de 15 à 60 ans, p = 0,00. Trois cent vingt trois actes étaient douloureux. Les gestes douloureux les plus fréquemment rapportés étaient les ponctions vasculaires dans 49,54% (n = 160) des cas (Tableau 1). L'intensité de la douleur était rapportée comme étant sévère à extrêmement sévère dans 17,34% (n = 20) des cas. L'intensité de la douleur augmentait lorsque les soins étaient répétés, p = 0,14. En effet, les patients ayant subi des soins répétés signalaient une douleur sévère à extrêmement sévère dans 19,18% (n = 21). Dans 61,76% des cas (n = 63), la douleur était maximale au moment de la réalisation des soins, p = 0,00. Les personnels paramédicaux effectuaient des soins douloureux dans 47,05% (n = 48) des cas. Cinquante sept pourcent des patients (n = 59) déclaraient avoir reçu une mesure préventive par une information verbale immédiatement avant l'acte. Les patients naïfs 60,71% (n = 61) étaient plus anxieux que les patients expérimentés 39,29% (n = 41), p = 0,0. Le transport par chaise roulante était très douloureux dans 83,34% (n = 85) des cas, suivi par la marche à pied dans 10,78% (n = 11) et transport au dos 5,88% (n = 6) des cas. Les patients gardaient de mauvais souvenir dans 64,71% (n = 66) des cas, p = 0,00. Ils avaient l'intention de refuser les soins dans 14,71% (n = 15), p = 0,00.

**Tableau 1:** Répartition des procédures douloureuses

Types de procédures	Nombre (n)	Pourcentage (%)
Ponctions vasculaires	160	49,54
Transports	48	14,86
Traitements par injection	40	12,38
Ponctions non vasculaires	38	11,76
Gestes à visée diagnostiques et thérapeutiques	17	5,27
Autres gestes invasifs	14	4,33
Autres soins thérapeutiques	6	1,86
Total	323	100

## Discussion

Cette étude avait montré une fréquence de 69,86% des douleurs induites pas les soins. Le genre féminin prédominait. La moyenne d’âge était de 46 ans. Les ponctions vasculaires provoquaient plus de douleur. Les infirmiers réalisaient ces actes. L'information verbale était le moyen préventif le plus utilisé. Les patients naïfs étaient très anxieux. Plus de la moitié des malades gardaient de mauvais souvenir.

La fréquence de douleurs induites par les soins dans notre travail était très élevée par rapport aux données des littératures. En France, elle varie de 19% à 55% [2, 3]. La fréquence des douleurs induites est variable selon les études possiblement en fonction de la méthodologie employée, de la pathologie des malades hospitalisés et de leurs caractéristiques [4]. A notre connaissance, cette étude représentait le premier travail à évaluer la douleur induite par les soins dans un échantillon de population Malgache adulte hospitalisé au CHU de Befelatanana. Notre série ne pourrait pas refléter les situations dans la population générale mais elle pourrait données des idées dans les autres structures hospitalières à Madagascar. Nous avions retrouvé une prédominance féminine, comparable aux données de la littérature [4]. Dans les données expérimentales, le seuil de perception de la douleur est plus bas chez la femme et pourrait exposer à une perception accrue des douleurs au cours des soins [5]. L’âge moyen de notre étude était de 46 ans. Dans certaines études, l’âge inférieur ou égal à 60 ans apparaît comme un facteur pouvant augmenter le ressenti de la douleur liée aux soins [6, 7]. Ces résultats traduisaient soit une plus grande sensibilité des sujets jeunes à la douleur soit la réticence des sujets plus âgés de parler leur douleur. Les ponctions vasculaires étaient les gestes les plus douloureux. Ces résultats sont similaires aux études réalisées au sein des trois Hôpitaux de Paris, avec une fréquence de la ponction vasculaire à 64% [8]. Par ailleurs, l'intensité de la douleur augmentait lorsque les soins étaient répétés. Nos donnés sont confirmées par la littérature [9]. La majorité des actes douloureux a été réalisée par les infirmiers dans 47,05% mais sans différence significative, similaire aux données de la littérature [3]. Cela pourrait s'expliquer par le fait que les infirmiers exécutaient des nombreux soins hospitaliers. Les infirmiers devaient envisager des méthodes pour réduire le nombre de répétition des soins. D'autant plus que même les patients ayant reçu une information verbale exprimaient aussi des douleurs. Au CHU de Grenoble, 43% ont déclaré avoir reçu une information sur la douleur avant le soin [2]. En effet, différentes études sur information et douleur montrent que selon la façon dont elle est faite, l'information peut soit diminuer, soit augmenter la douleur ressentie au cours du geste [4]. Par conséquent, former les soignants sur la façon de communiquer avec les patients pourrait donc être bénéfique et diminuer les douleurs induites. En plus, les patients naïfs étaient plus anxieux que les patients expérimentés. Ce résultat va dans le sens des études qui montrent qu'une plus grande anxiété entraîne une plus grande douleur lors du geste [4]. Certains patients s’étaient déplacés à pied et au dos vers le service de destination. Notre travail serait la première à aborder un problème méconnu et qui, pourtant, concernait quotidiennement les patients prise en charge dans une structure hospitalière. Ces patients avaient une idée de refuser les soins dans 14,71% des cas du fait probablement de soins qualifiés potentiellement douloureux.

## Conclusion

Cette étude avait montré la fréquence inattendue des actes douloureux. Elle avait montré également que de nombreux soins considérés comme routine et sans danger étaient potentiellement douloureux. En effet, la douleur reste toujours un souvenir pénible pour le patient même si les soins ont par ailleurs été efficients. Une amélioration de la prévention de cette douleur induite serait nécessaire pour faciliter l'adhésion des patients aux différents soins hospitaliers et pour augmenter le taux de fréquentation hospitalière dans des pays où la médecine traditionnelle garde encore une place importante.
